# Draft genomes of *Aeromonas* species isolated from humans

**DOI:** 10.1128/mra.00757-25

**Published:** 2025-10-21

**Authors:** Jane M. Michalski, Bernadette A. Hritzo, Jennifer A. Jones, Jasnehta Permala-Booth, Sharon M. Tennant, David A. Rasko

**Affiliations:** 1 Institute for Genome Sciences, University of Maryland School of Medicine 189800, Baltimore, Maryland, USA; 2 Department of Microbiology and Immunology, University of Maryland School of Medicine 200790, Baltimore, Maryland, USA; 3 Center for Pathogen Research, University of Maryland School of Medicine 12264, Baltimore, Maryland, USA; 4 Center for Vaccine Development and Global Health, University of Maryland School of Medicine 197255, Baltimore, Maryland, USA; 5 Department of Medicine, University of Maryland School of Medicine 195439, Baltimore, Maryland, USA; 6 Department of Microbial Pathogenesis, University of Maryland School of Dentistry 89015, Baltimore, Maryland, USA; Wellesley College, Wellesley, Massachusetts, USA

**Keywords:** *Aeromonas*, *Aeromonas caviae*, human, genome

## Abstract

*Aeromonas* isolates are rarely speciated, resulting in a lack of understanding of the species distribution among human infections. In this study, we describe the genomes of 431 *Aeromonas* isolates associated with human colonization and infection, providing insight into the species distribution associated with human infection.

## ANNOUNCEMENT

The *Aeromonas* genus contains more than thirty species, but only four species, *A. hydrophila*, *A. veronii*, *A. dhakensis*, and *A. caviae*, have been associated with significant human infection (1, 2). Human infections caused by *Aeromonas* spp. are increasing and have been associated with multiple clinical syndromes including, but not limited to gastroenteritis, cellulitis, meningitis, and pneumonia in both immunocompetent and immunocompromised hosts (3–8). In this study, we generated draft genomes of 431 *Aeromonas* spp. isolates obtained as part of the Global Enteric Multicenter Study (GEMS) (6, 9).

The *Aeromonas* spp. isolates were initially isolated as described in Panchalingam et al. (10). Briefly, feces or a rectal swab were transported in cold containers and inoculated into transport media within 6 h; samples were inoculated into selective growth media for multiple pathogens within 18 h from collection (10). For *Aeromonas* spp., Ryan agar plates incubated for 48 h at 35–37 °C were examined for dark green colonies with darker green centers. Presumptive *Aeromonas* spp. colonies were sub-cultured on trypticase soy agar (TSA) overnight at 37°C. Single colonies were then cultured aerobically at 37°C for 24 h on TSA agar with differing concentrations of NaCl (0%, 6%, and 8%) to assess salt tolerance. The isolates were further examined by biochemical assays including oxidase and catalase tests. Susceptibility to 2, 4-diamino-6, 7-diisopropyl pteridine (O/129) was assessed by the addition of a 150 µg disc to TSA (11). Any oxidase- and catalase-positive isolate that grew in 0% NaCl but not in 6% or 8%, and was O/129 resistant was designated an *Aeromonas* spp. isolate. *Aeromonas* spp. isolates were frozen in 20% glycerol and maintained at −80°C at the Center for Vaccine Development and Global Health at the University of Maryland School of Medicine.

Isolates were resurrected from frozen culture on Lysogeny agar overnight at 37°C and genomic DNA was isolated using the MagAttract Power Microbiome DNA/RNA Kit (Qiagen) automated on a Hamilton Microlab STAR robotic platform. Paired-end sequencing libraries were generated with unsheared genomic DNA using the Kapa library kit (Roche), then sequenced with 2×150  bp chemistry on the Illumina HiSeq 4000 platform. Illumina reads were trimmed for quality using Trimmomatic v.0.36 (12) and assembled using SPAdes v.3.11.1 with careful mismatch correction (13). All software was run with default values. The total number of reads generated for each isolate, relevant sequencing statistics, and GenBank accession numbers of each assembly are provided in Table 1. The average number of reads generated in this study was 676,675 reads (range 279,2036–1,554,5206), resulting in an average coverage value of 230× (range 94–544×). The assembled genomes contained, on average, 87 contigs (range 26–347 contigs), with an average genome size of 4,450,999 bp (range 4,011,918–4,959,356 bp), and an average GC% of 60.62% (range 58.31–63.04%). The genome sequence was used for speciation based on phylogenomic relatedness to reference genomes.

**TABLE 1:** *Aeromonas* species isolate data

	Data for isolates				Genomic statistics for each isolate								Genbank accession numbers		
Species	Isolate	Isolate	Country	Type	Number of reads	Number of bases	Coverage	No. of Contigs	*N*_50_	Size (bp)	% GC	ST	Biosample	SRA	Assembly
*Aeromonas caviae*	A500297	500297	India	Case	7,255,376	1,095,561,776	248.0	61	146,810	4,417,741	61.4	1,471	SAMN14146648	SRR11247485	JAAKWL000000000
*Aeromonas caviae*	A503237	503237	India	Case	6,880,560	1,038,964,560	231.7	58	156,263	4,484,452	61.82	1,521	SAMN14146650	SRR11247091	JAAKWJ000000000
*Aeromonas caviae*	A600056	600056	Bangladesh	Case	3,281,570	495,517,070	113.7	39	253,195	4,359,004	62.08	1,589	SAMN14147056	SRR11247313	JAAKXJ000000000
*Aeromonas caviae*	A600142	600142	Bangladesh	Case	3,942,282	595,284,582	136.1	53	186,580	4,375,045	61.81	592	SAMN14146657	SRR11247484	JAAKWC000000000
*Aeromonas caviae*	A600199	600199	Bangladesh	Case	3,944,440	595,610,440	136.2	68	159,194	4,374,400	61.88	1,480	SAMN14146661	SRR11247442	JAAKVY000000000
*Aeromonas caviae*	A600239	600239	Bangladesh	Case	4,000,770	604,116,270	133.5	108	80,105	4,525,065	61.39	1,581	SAMN14147058	SRR11247310	JAAKXH000000000
*Aeromonas caviae*	A600241	600241	Bangladesh	Case	3,905,484	589,728,084	133.3	98	97,527	4,423,934	61.54	181	SAMN14147059	SRR11247309	JAAKXG000000000
*Aeromonas caviae*	A600277	600277	Bangladesh	Case	3,344,158	504,967,858	118.6	50	171,235	4,257,909	61.94	1,519	SAMN14146666	SRR11247215	JAAKVT000000000
*Aeromonas caviae*	A600295	600295	Bangladesh	Case	4,376,974	660,923,074	156.3	100	101,595	4,228,291	61.83	1,468	SAMN14146669	SRR11247181	JAAKVQ000000000
*Aeromonas caviae*	A600334	600334	Bangladesh	Case	2,993,178	451,969,878	103.2	51	154,451	4,378,983	61.84	1,510	SAMN14146671	SRR11247157	JAAKVO000000000
*Aeromonas caviae*	A600358	600358	Bangladesh	Case	3,611,200	545,291,200	121.8	52	146,608	4,478,117	61.84	1,522	SAMN14146674	SRR11247124	JAAKVL000000000
*Aeromonas caviae*	A600406	600406	Bangladesh	Case	6,702,098	1,012,016,798	236.1	89	105,920	4,285,580	61.73	1,465	SAMN14146676	SRR11247102	JAAKVJ000000000
*Aeromonas caviae*	A600430	600430	Bangladesh	Case	3,737,212	564,319,012	129.4	54	161,294	4,362,173	62.06	1,559	SAMN14146677	SRR11247090	JAAKVI000000000
*Aeromonas caviae*	A600480	600480	Bangladesh	Case	3,541,654	534,789,754	123.5	50	167,108	4,330,534	61.98	1,560	SAMN14147061	SRR11247307	JAAKXE000000000
*Aeromonas caviae*	A600518	600518	Bangladesh	Case	2,792,036	421,597,436	94.5	142	70,780	4,462,279	61.42	1,263	SAMN14146683	SRR11247368	JAAKVC000000000
*Aeromonas caviae*	A600521	600521	Bangladesh	Case	3,556,666	537,056,566	124.7	75	123,303	4,307,251	61.91	1,525	SAMN14146684	SRR11247357	JAAKVB000000000
*Aeromonas caviae*	A600558	600558	Bangladesh	Case	3,489,160	526,863,160	124.0	72	117,570	4,249,059	61.78	1,545	SAMN14146687	SRR11247323	JAAKUY000000000
*Aeromonas caviae*	A600566	600566	Bangladesh	Case	2,963,934	447,554,034	103.7	49	166,393	4,315,236	61.81	1,472	SAMN14147063	SRR11247305	JAAKXC000000000
*Aeromonas caviae*	A600673	600673	Bangladesh	Case	3,369,702	508,825,002	114.7	47	215,889	4,436,909	61.97	1,557	SAMN14147065	SRR11247303	JAAKXA000000000
*Aeromonas caviae*	A600716	600716	Bangladesh	Case	3,485,068	526,245,268	121.9	61	182,543	4,317,007	61.98	1,564	SAMN14147066	SRR11247302	JAAKWZ000000000
*Aeromonas caviae*	A600744	600744	Bangladesh	Case	3,291,950	497,084,450	115.2	85	132,714	4,315,918	61.68	1,543	SAMN14146700	SRR11247277	JAAKUL000000000
*Aeromonas caviae*	A600749	600749	Bangladesh	Case	3,991,728	602,750,928	139.6	94	104,240	4,317,000	61.55	1,465	SAMN14147068	SRR11247299	JAAKWX000000000
*Aeromonas caviae*	A600856	600856	Bangladesh	Case	8,519,626	1,286,463,526	298.3	73	172,753	4,313,120	61.84	1,488	SAMN14146708	SRR11247268	JAAKUD000000000
*Aeromonas caviae*	A600866	600866	Bangladesh	Case	6,445,688	973,298,888	223.0	84	124,553	4,363,622	61.78	1,491	SAMN14146709	SRR11247267	JAAKUC000000000
*Aeromonas caviae*	A600910	600910	Bangladesh	Case	6,506,530	982,486,030	226.8	66	130,066	4,332,475	61.88	1,547	SAMN14146711	SRR11247265	JAAKUA000000000
*Aeromonas caviae*	A600919	600919	Bangladesh	Case	3,669,758	554,133,458	129.7	51	194,030	4,271,710	61.92	1,498	SAMN14147070	SRR11247297	JAAKWV000000000
*Aeromonas caviae*	A600932	600932	Bangladesh	Case	7,095,314	1,071,392,414	250.9	62	136,356	4,269,971	61.84	1,505	SAMN14146712	SRR11247264	JAAKTZ000000000
*Aeromonas caviae*	A600960	600960	Bangladesh	Case	7,723,230	1,166,207,730	262.0	41	263,617	4,451,166	62.09	1,548	SAMN14146714	SRR11247262	JAAKTX000000000
*Aeromonas caviae*	A600965	600965	Bangladesh	Case	6,564,072	991,174,872	229.9	98	103,431	4,312,004	61.56	1,550	SAMN14146716	SRR11247260	JAAKTV000000000
*Aeromonas caviae*	A600973	600973	Bangladesh	Case	4,870,190	735,398,690	166.9	37	198,042	4,404,910	61.83	1,437	SAMN14146718	SRR11247257	JAAKTT000000000
*Aeromonas caviae*	A600989	600989	Bangladesh	Case	3,896,864	588,426,464	135.6	38	239,242	4,339,751	61.77	1,551	SAMN14146721	SRR11247254	JAAKTQ000000000
*Aeromonas caviae*	A601060	601060	Bangladesh	Case	7,260,120	1,096,278,120	252.4	69	114,786	4,343,099	61.78	1,444	SAMN14146729	SRR11247505	JAAKTI000000000
*Aeromonas caviae*	A601085	601085	Bangladesh	Case	6,403,852	966,981,652	222.7	50	174,095	4,341,390	62.01	1,436	SAMN14146732	SRR11247502	JAAKTF000000000
*Aeromonas caviae*	A601149	601149	Bangladesh	Case	4,042,836	610,468,236	142.0	48	214,514	4,300,582	61.85	1,493	SAMN14146739	SRR11247494	JAAKSY000000000
*Aeromonas caviae*	A601294	601294	Bangladesh	Case	2,914,082	440,026,382	100.9	127	67,330	4,362,463	61.95	479	SAMN14146747	SRR11247483	JAAKSQ000000000
*Aeromonas caviae*	A602018	602018	Bangladesh	Case	5,971,458	901,690,158	206.9	45	199,347	4,357,361	61.82	1,554	SAMN14146749	SRR11247481	JAAKSO000000000
*Aeromonas caviae*	A602193	602193	Bangladesh	Case	7,608,524	1,148,887,124	261.0	167	53,584	4,401,376	61.33	313	SAMN14146751	SRR11247479	JAAKSM000000000
*Aeromonas caviae*	A602312	602312	Bangladesh	Case	7,791,132	1,176,460,932	268.5	83	131,395	4,381,875	61.56	1,463	SAMN14146758	SRR11247471	JAAKSF000000000
*Aeromonas caviae*	A602429	602429	Bangladesh	Case	6,249,544	943,681,144	216.7	86	112,720	4,354,756	61.53	1,539	SAMN14146762	SRR11247467	JAAKSB000000000
*Aeromonas caviae*	A602432	602432	Bangladesh	Case	7,687,630	1,160,832,130	262.2	50	172,235	4,427,214	61.73	1,555	SAMN14146763	SRR11247466	JAAKSA000000000
*Aeromonas caviae*	A602434	602434	Bangladesh	Case	7,522,260	1,135,861,260	264.6	98	102,693	4,292,695	61.73	1,556	SAMN14146764	SRR11247465	JAAKRZ000000000
*Aeromonas caviae*	A602438	602438	Bangladesh	Case	7,804,816	1,178,527,216	274.6	86	115,676	4,292,351	61.84	1,537	SAMN14146765	SRR11247464	JAAKRY000000000
*Aeromonas caviae*	A602443	602443	Bangladesh	Case	8,419,166	1,271,294,066	295.0	59	173,057	4,308,879	61.77	1,558	SAMN14146766	SRR11247463	JAAKRX000000000
*Aeromonas caviae*	A602491	602491	Bangladesh	Case	8,106,724	1,224,115,324	280.7	79	100,838	4,361,291	61.94	1,561	SAMN14146770	SRR11247170	JAAKRT000000000
*Aeromonas caviae*	A602497	602497	Bangladesh	Case	7,538,984	1,138,386,584	264.9	44	209,749	4,298,164	62.07	1,562	SAMN14146771	SRR11247169	JAAKRS000000000
*Aeromonas caviae*	A602511	602511	Bangladesh	Case	7,057,406	1,065,668,306	252.5	84	100,571	4,220,619	62.02	1,494	SAMN14146772	SRR11247458	JAAKRR000000000
*Aeromonas caviae*	A602512	602512	Bangladesh	Case	6,579,792	993,548,592	203.2	131	88,779	4,890,330	60.48	1,515	SAMN14146773	SRR11247457	JAAKRQ000000000
*Aeromonas caviae*	A602521	602521	Bangladesh	Case	7,305,854	1,103,183,954	260.4	95	97,083	4,236,432	61.79	1,563	SAMN14146774	SRR11247456	JAAKRP000000000
*Aeromonas caviae*	A602596	602596	Bangladesh	Case	8,151,346	1,230,853,246	278.3	230	38,115	4,422,984	61.44	1,506	SAMN14146778	SRR11247451	JAAKRL000000000
*Aeromonas caviae*	A602607	602607	Bangladesh	Case	6,551,922	989,340,222	224.8	172	57,889	4,401,455	61.38	1,586	SAMN14146780	SRR11247449	JAAKRJ000000000
*Aeromonas caviae*	A602614	602614	Bangladesh	Case	8,897,092	1,343,460,892	311.4	68	157,106	4,314,670	61.78	1,538	SAMN14146781	SRR11247448	JAAKRI000000000
*Aeromonas caviae*	A602682	602682	Bangladesh	Case	10,834,440	1,636,000,440	348.7	121	125,667	4,691,549	61.14	1,513	SAMN14146785	SRR11247444	JAAKRE000000000
*Aeromonas caviae*	A602686	602686	Bangladesh	Case	7,745,972	1,169,641,772	265.3	85	130,772	4,409,423	61.69	1,470	SAMN14146786	SRR11247443	JAAKRD000000000
*Aeromonas caviae*	A602710	602710	Bangladesh	Case	6,560,654	990,658,754	223.8	170	54,097	4,426,443	61.55	1,475	SAMN14146787	SRR11247441	JAAKRC000000000
*Aeromonas caviae*	A602747	602747	Bangladesh	Case	8,422,364	1,271,776,964	287.6	102	97,908	4,422,199	61.73	1,565	SAMN14146788	SRR11247440	JAAKRB000000000
*Aeromonas caviae*	A602762	602762	Bangladesh	Case	4,718,520	712,496,520	163.0	118	68,181	4,371,576	61.37	1,529	SAMN14146791	SRR11247437	JAAKQY000000000
*Aeromonas caviae*	A602763	602763	Bangladesh	Case	6,789,948	1,025,282,148	234.4	113	86,295	4,374,540	61.58	1,416	SAMN14146792	SRR11247436	JAAKQX000000000
*Aeromonas caviae*	A602764	602764	Bangladesh	Case	6,422,800	969,842,800	227.3	71	121,639	4,266,139	61.78	1,568	SAMN14146793	SRR11247435	JAAKQW000000000
*Aeromonas caviae*	A602765	602765	Bangladesh	Case	5,448,872	822,779,672	187.9	112	81,465	4,378,878	61.7	1,474	SAMN14146794	SRR11247434	JAAKQV000000000
*Aeromonas caviae*	A602768	602768	Bangladesh	Case	7,008,438	1,058,274,138	243.9	63	137,264	4,338,913	61.77	1,518	SAMN14146796	SRR11247432	JAAKQT000000000
*Aeromonas caviae*	A602784	602784	Bangladesh	Case	6,423,996	970,023,396	222.6	53	179,177	4,358,280	61.96	1,569	SAMN14146797	SRR11247430	JAAKQS000000000
*Aeromonas caviae*	A602809	602809	Bangladesh	Case	5,499,226	830,383,126	192.9	61	176,528	4,304,327	61.74	1,486	SAMN14146798	SRR11247429	JAAKQR000000000
*Aeromonas caviae*	A602854	602854	Bangladesh	Case	8,434,456	1,273,602,856	291.6	153	65,518	4,367,715	61.41	313	SAMN14146800	SRR11247427	JAAKQP000000000
*Aeromonas caviae*	A602900	602900	Bangladesh	Case	6,465,028	976,219,228	211.8	161	73,930	4,609,974	60.91	1,450	SAMN14146802	SRR11247425	JAAKQN000000000
*Aeromonas caviae*	A602905	602905	Bangladesh	Case	5,717,358	863,321,058	198.5	88	118,118	4,348,587	61.87	1,454	SAMN14146803	SRR11247424	JAAKQM000000000
*Aeromonas caviae*	A602931	602931	Bangladesh	Case	7,673,646	1,158,720,546	242.4	142	79,671	4,779,582	60.44	313	SAMN14146806	SRR11247249	JAAKQJ000000000
*Aeromonas caviae*	A602962	602962	Bangladesh	Case	5,619,886	848,602,786	199.0	85	112,673	4,264,637	61.87	1,570	SAMN14146808	SRR11247246	JAAKQH000000000
*Aeromonas caviae*	A602985	602985	Bangladesh	Case	9,171,132	1,384,840,932	318.8	44	172,449	4,344,538	62.03	1,572	SAMN14146809	SRR11247245	JAAKQG000000000
*Aeromonas caviae*	A603039	603039	Bangladesh	Case	9,586,858	1,447,615,558	329.1	47	205,745	4,398,834	61.85	1,481	SAMN14146813	SRR11247241	JAAKQC000000000
*Aeromonas caviae*	A603040	603040	Bangladesh	Case	6,254,136	944,374,536	208.5	52	217,396	4,528,550	61.69	94	SAMN14146814	SRR11247240	JAAKQB000000000
*Aeromonas caviae*	A603041	603041	Bangladesh	Case	7,673,358	1,158,677,058	265.2	112	101,038	4,368,513	61.45	1,251	SAMN14146815	SRR11247239	JAAKQA000000000
*Aeromonas caviae*	A603047	603047	Bangladesh	Case	6,340,374	957,396,474	196.1	174	58,103	4,882,880	60.17	1,524	SAMN14146816	SRR11247238	JAAKPZ000000000
*Aeromonas caviae*	A603114	603114	Bangladesh	Case	7,562,070	1,141,872,570	257.3	200	43,529	4,437,277	61.31	1,449	SAMN14146821	SRR11247232	JAAKPU000000000
*Aeromonas caviae*	A603171	603171	Bangladesh	Case	8,488,614	1,281,780,714	297.4	57	139,582	4,310,188	61.83	1,497	SAMN14146822	SRR11247231	JAAKPT000000000
*Aeromonas caviae*	A603246	603246	Bangladesh	Case	6,024,142	909,645,442	208.1	78	119,140	4,371,553	61.69	1,573	SAMN14146825	SRR11247228	JAAKPQ000000000
*Aeromonas caviae*	A603253	603253	Bangladesh	Case	5,963,916	900,551,316	206.5	153	70,596	4,360,359	61.61	313	SAMN14146826	SRR11247227	JAAKPP000000000
*Aeromonas caviae*	A603287	603287	Bangladesh	Case	7,220,932	1,090,360,732	244.4	117	88,791	4,460,570	61.44	1,441	SAMN14146828	SRR11247224	JAAKPN000000000
*Aeromonas caviae*	A603360	603360	Bangladesh	Case	5,756,696	869,261,096	205.3	62	126,508	4,233,753	61.95	1,526	SAMN14146832	SRR11247220	JAAKPJ000000000
*Aeromonas caviae*	A603392	603392	Bangladesh	Case	7,037,946	1,062,729,846	245.9	178	51,026	4,321,924	61.78	1,460	SAMN14146835	SRR11247217	JAAKPG000000000
*Aeromonas caviae*	A603465	603465	Bangladesh	Case	6,530,462	986,099,762	227.2	49	188,119	4,340,241	62.01	1,574	SAMN14146838	SRR11247213	JAAKPD000000000
*Aeromonas caviae*	A603573	603573	Bangladesh	Case	7,860,042	1,186,866,342	276.2	45	205,581	4,297,547	61.97	940	SAMN14146842	SRR11247209	JAAKOZ000000000
*Aeromonas caviae*	A603579	603579	Bangladesh	Case	6,602,802	997,023,102	223.4	255	38,267	4,461,966	61.55	1,514	SAMN14146843	SRR11247208	JAAKOY000000000
*Aeromonas caviae*	A603595	603595	Bangladesh	Case	6,943,366	1,048,448,266	237.5	105	98,142	4,414,349	61.48	1,530	SAMN14146844	SRR11247207	JAAKOX000000000
*Aeromonas caviae*	A603625	603625	Bangladesh	Case	8,216,084	1,240,628,684	288.7	153	55,590	4,297,237	61.8	1,469	SAMN14146849	SRR11247200	JAAKOS000000000
*Aeromonas caviae*	A603650	603650	Bangladesh	Case	7,665,444	1,157,482,044	266.0	165	57,234	4,351,534	61.6	1,263	SAMN14146851	SRR11247198	JAAKOQ000000000
*Aeromonas caviae*	A603688	603688	Bangladesh	Case	6,419,708	969,375,908	222.9	61	182,389	4,348,054	61.82	1,575	SAMN14146855	SRR11247194	JAAKOM000000000
*Aeromonas caviae*	A603690	603690	Bangladesh	Case	6,842,660	1,033,241,660	208.3	151	65,365	4,959,356	59.47	1,576	SAMN14146856	SRR11247193	JAAKOL000000000
*Aeromonas caviae*	A603761	603761	Bangladesh	Case	7,783,362	1,175,287,662	266.9	34	222,887	4,403,559	61.99	1,482	SAMN14146865	SRR11247183	JAAKOC000000000
*Aeromonas caviae*	A603822	603822	Bangladesh	Case	7,720,360	1,165,774,360	267.0	161	59,441	4,366,041	61.54	1,578	SAMN14146866	SRR11247182	JAAKOB000000000
*Aeromonas caviae*	A603869	603869	Bangladesh	Case	7,020,698	1,060,125,398	240.3	40	225,244	4,411,063	61.82	1,517	SAMN14146867	SRR11247180	JAAKOA000000000
*Aeromonas caviae*	A603899	603899	Bangladesh	Case	8,598,226	1,298,332,126	295.4	39	204,773	4,394,823	62	1,579	SAMN14146868	SRR11247179	JAAKNZ000000000
*Aeromonas caviae*	A603901	603901	Bangladesh	Case	6,923,984	1,045,521,584	236.8	180	55,291	4,415,727	61.27	1,534	SAMN14146869	SRR11247178	JAAKNY000000000
*Aeromonas caviae*	A603962	603962	Bangladesh	Case	7,792,068	1,176,602,268	271.2	57	177,646	4,338,135	61.78	1,611	SAMN14146872	SRR11247175	JAAKNV000000000
*Aeromonas caviae*	A604001	604001	Bangladesh	Case	5,698,996	860,548,396	199.8	76	137,968	4,307,380	61.61	1,489	SAMN14146874	SRR11247173	JAAKNT000000000
*Aeromonas caviae*	A604002	604002	Bangladesh	Case	6,289,738	949,750,438	222.5	65	123,535	4,269,236	61.87	1,612	SAMN14146875	SRR11247172	JAAKNS000000000
*Aeromonas caviae*	A604022	604022	Bangladesh	Case	7,155,202	1,080,435,502	249.7	81	120,357	4,326,196	61.86	479	SAMN14146878	SRR11247166	JAAKNP000000000
*Aeromonas caviae*	A604053	604053	Bangladesh	Case	6,672,368	1,007,527,568	225.0	173	60,288	4,477,967	61.38	1,447	SAMN14146879	SRR11247165	JAAKNO000000000
*Aeromonas caviae*	A604054	604054	Bangladesh	Case	6,397,362	966,001,662	224.2	85	107,650	4,307,857	61.56	1,465	SAMN14146880	SRR11247164	JAAKNN000000000
*Aeromonas caviae*	A604074	604074	Bangladesh	Case	8,151,178	1,230,827,878	281.4	53	185,642	4,374,109	61.94	1,580	SAMN14146881	SRR11247163	JAAKNM000000000
*Aeromonas caviae*	A604081	604081	Bangladesh	Case	7,422,786	1,120,840,686	250.7	84	136,842	4,471,477	61.48	1,495	SAMN14146882	SRR11247162	JAAKNL000000000
*Aeromonas caviae*	A604096	604096	Bangladesh	Case	6,629,062	1,000,988,362	230.4	99	79,624	4,344,975	61.58	1,500	SAMN14146884	SRR11247160	JAAKNJ000000000
*Aeromonas caviae*	A604124	604124	Bangladesh	Case	5,699,876	860,681,276	187.6	117	80,105	4,587,819	61.31	1,581	SAMN14146885	SRR11247159	JAAKNI000000000
*Aeromonas caviae*	A604136	604136	Bangladesh	Case	6,589,164	994,963,764	219.1	134	92,011	4,540,285	61.39	1,491	SAMN14146886	SRR11247158	JAAKNH000000000
*Aeromonas caviae*	A604141	604141	Bangladesh	Case	7,566,796	1,142,586,196	253.8	241	44,816	4,501,661	61.3	1,476	SAMN14146887	SRR11247156	JAAKNG000000000
*Aeromonas caviae*	A604142	604142	Bangladesh	Case	7,215,446	1,089,532,346	246.3	168	58,084	4,423,136	61.35	1,448	SAMN14146888	SRR11247155	JAAKNF000000000
*Aeromonas caviae*	A604185	604185	Bangladesh	Case	6,729,876	1,016,211,276	234.5	74	99,001	4,333,426	61.84	1,582	SAMN14146890	SRR11247153	JAAKND000000000
*Aeromonas caviae*	A604193	604193	Bangladesh	Case	10,506,664	1,586,506,264	353.3	201	47,793	4,490,888	61.39	313	SAMN14146891	SRR11247152	JAAKNC000000000
*Aeromonas caviae*	A604281	604281	Bangladesh	Case	6,904,670	1,042,605,170	228.8	161	61,092	4,557,697	61.25	1,509	SAMN14146897	SRR11247145	JAAKMW000000000
*Aeromonas caviae*	A604295	604295	Bangladesh	Case	5,711,726	862,470,626	200.2	83	96,715	4,308,552	61.84	1,567	SAMN14146899	SRR11247143	JAAKMU000000000
*Aeromonas caviae*	A604333	604333	Bangladesh	Case	7,015,344	1,059,316,944	239.9	95	97,444	4,415,200	61.4	1,542	SAMN14146901	SRR11247141	JAAKMS000000000
*Aeromonas caviae*	A604339	604339	Bangladesh	Case	6,610,386	998,168,286	204.6	347	36,968	4,879,292	61.02	1,584	SAMN14146902	SRR11247140	JAAKMR000000000
*Aeromonas caviae*	A604370	604370	Bangladesh	Case	7,696,020	1,162,099,020	273.3	63	151,898	4,252,503	61.96	1,504	SAMN14146904	SRR11247138	JAAKMP000000000
*Aeromonas caviae*	A604500	604500	Bangladesh	Case	8,611,116	1,300,278,516	288.5	129	82,247	4,507,087	61.52	1,487	SAMN14146918	SRR11247122	JAAKMB000000000
*Aeromonas caviae*	A604504	604504	Bangladesh	Case	8,797,390	1,328,405,890	308.8	40	225,390	4,301,841	62.16	1,588	SAMN14146919	SRR11247121	JAAKMA000000000
*Aeromonas caviae*	A604518	604518	Bangladesh	Case	6,616,256	999,054,656	230.4	103	109,754	4,335,689	61.65	1,512	SAMN14146920	SRR11247120	JAAKLZ000000000
*Aeromonas caviae*	A604533	604533	Bangladesh	Case	8,311,280	1,255,003,280	287.2	42	163,266	4,370,204	61.77	1,473	SAMN14146921	SRR11247119	JAAKLY000000000
*Aeromonas caviae*	A700004	700004	Pakistan	Case	6,717,478	1,014,339,178	231.4	139	70,342	4,382,676	61.44	1,263	SAMN14146924	SRR11247116	JAAKLV000000000
*Aeromonas caviae*	A700006	700006	Pakistan	Case	5,425,994	819,325,094	184.2	175	65,348	4,447,357	61.28	1,453	SAMN14146925	SRR11247115	JAAKLU000000000
*Aeromonas caviae*	A700049	700049	Pakistan	Case	8,599,970	1,298,595,470	290.6	93	123,941	4,468,654	61.41	1,461	SAMN14146928	SRR11247111	JAAKLR000000000
*Aeromonas caviae*	A700053	700053	Pakistan	Case	9,877,056	1,491,435,456	332.3	139	58,872	4,487,753	61.34	313	SAMN14146930	SRR11247109	JAAKLP000000000
*Aeromonas caviae*	A700092	700092	Pakistan	Case	8,434,978	1,273,681,678	289.3	125	71,969	4,402,728	61.54	1,443	SAMN14146933	SRR11247106	JAAKLM000000000
*Aeromonas caviae*	A700144	700144	Pakistan	Case	7,125,776	1,075,992,176	251.8	63	127,247	4,273,066	61.96	1,502	SAMN14146934	SRR11247105	JAAKLL000000000
*Aeromonas caviae*	A700172	700172	Pakistan	Case	7,357,420	1,110,970,420	253.4	34	247,134	4,384,247	62.01	1,591	SAMN14146935	SRR11247104	JAAKLK000000000
*Aeromonas caviae*	A700239	700239	Pakistan	Case	4,465,740	674,326,740	152.0	307	25,007	4,436,167	61.38	313	SAMN14146937	SRR11247101	JAAKLI000000000
*Aeromonas caviae*	A700241	700241	Pakistan	Case	7,821,618	1,181,064,318	269.7	35	234,332	4,379,140	61.99	1,592	SAMN14146938	SRR11247100	JAAKLH000000000
*Aeromonas caviae*	A700329	700329	Pakistan	Case	7,326,042	1,106,232,342	252.3	203	51,481	4,385,255	61.61	1,594	SAMN14146941	SRR11247097	JAAKLE000000000
*Aeromonas caviae*	A700335	700335	Pakistan	Case	9,313,834	1,406,388,934	320.3	103	95,887	4,390,799	61.63	1,532	SAMN14146942	SRR11247096	JAAKLD000000000
*Aeromonas caviae*	A700339	700339	Pakistan	Case	9,591,034	1,448,246,134	335.5	55	147,982	4,316,142	61.96	1,438	SAMN14146943	SRR11247095	JAAKLC000000000
*Aeromonas caviae*	A700380	700380	Pakistan	Case	9,529,862	1,439,009,162	330.3	312	27,722	4,357,307	61.8	1,613	SAMN14146946	SRR11247092	JAAKKZ000000000
*Aeromonas caviae*	A700394	700394	Pakistan	Case	7,228,886	1,091,561,786	248.5	205	40,714	4,392,105	61.36	1,587	SAMN14146948	SRR11247088	JAAKKX000000000
*Aeromonas caviae*	A700481	700481	Pakistan	Case	5,047,724	762,206,324	173.7	127	66,838	4,387,542	61.63	1,532	SAMN14146949	SRR11247087	JAAKKW000000000
*Aeromonas caviae*	A700493	700493	Pakistan	Case	6,747,106	1,018,813,006	235.5	92	104,000	4,326,058	61.82	1,613	SAMN14146951	SRR11247085	JAAKKU000000000
*Aeromonas caviae*	A700684	700684	Pakistan	Case	6,670,250	1,007,207,750	230.2	99	106,350	4,375,709	61.49	1,457	SAMN14146956	SRR11247080	JAAKKP000000000
*Aeromonas caviae*	A700701	700701	Pakistan	Case	8,286,480	1,251,258,480	283.7	127	83,212	4,411,228	61.29	1,540	SAMN14146959	SRR11247420	JAAKKM000000000
*Aeromonas caviae*	A700762	700762	Pakistan	Case	6,922,724	1,045,331,324	236.9	128	81,772	4,412,974	61.28	1,540	SAMN14146961	SRR11247418	JAAKKK000000000
*Aeromonas caviae*	A700783	700783	Pakistan	Case	7,785,916	1,175,673,316	270.9	74	124,131	4,340,496	61.93	1,501	SAMN14146962	SRR11247417	JAAKKJ000000000
*Aeromonas caviae*	A700818	700818	Pakistan	Case	7,933,242	1,197,919,542	269.4	151	68,525	4,446,906	61.24	1,523	SAMN14146965	SRR11247414	JAAKKG000000000
*Aeromonas caviae*	A700868	700868	Pakistan	Case	4,496,252	678,934,052	153.7	107	107,167	4,417,209	61.51	1,251	SAMN14147075	SRR11247292	JAAKWQ000000000
*Aeromonas caviae*	A702008	702008	Pakistan	Case	8,504,404	1,284,165,004	290.9	40	234,446	4,413,968	61.94	1,596	SAMN14146967	SRR11247411	JAAKKE000000000
*Aeromonas caviae*	A702050	702050	Pakistan	Case	9,451,612	1,427,193,412	322.2	54	186,712	4,429,455	62	1,597	SAMN14146968	SRR11247410	JAAKKD000000000
*Aeromonas caviae*	A702051	702051	Pakistan	Case	6,895,096	1,041,159,496	233.4	153	62,866	4,459,941	61.52	313	SAMN14146969	SRR11247409	JAAKKC000000000
*Aeromonas caviae*	A702154	702154	Pakistan	Case	8,560,576	1,292,646,976	291.0	160	61,290	4,442,532	61.26	1,534	SAMN14146972	SRR11247406	JAAKJZ000000000
*Aeromonas caviae*	A702186	702186	Pakistan	Case	7,124,588	1,075,812,788	238.6	208	54,060	4,509,400	61.4	1,598	SAMN14146973	SRR11247405	JAAKJY000000000
*Aeromonas caviae*	A702210	702210	Pakistan	Case	6,187,332	934,287,132	212.2	45	223,768	4,402,246	61.93	1,484	SAMN14146974	SRR11247404	JAAKJX000000000
*Aeromonas caviae*	A702252	702252	Pakistan	Case	3,364,964	508,109,564	118.5	85	106,789	4,289,479	61.91	1,496	SAMN14147076	SRR11247291	JAAKWP000000000
*Aeromonas caviae*	A702277	702277	Pakistan	Case	4,609,584	696,047,184	157.4	150	82,829	4,422,692	61.42	1,544	SAMN14146975	SRR11247403	JAAKJW000000000
*Aeromonas caviae*	A702278	702278	Pakistan	Case	7,713,996	1,164,813,396	258.2	210	54,060	4,510,489	61.41	1,598	SAMN14146976	SRR11247402	JAAKJV000000000
*Aeromonas caviae*	A702287	702287	Pakistan	Case	5,939,042	896,795,342	200.9	96	101,771	4,464,853	61.32	1,533	SAMN14146977	SRR11247400	JAAKJU000000000
*Aeromonas caviae*	A702332	702332	Pakistan	Case	7,346,016	1,109,248,416	247.8	52	184,972	4,476,073	61.78	1,601	SAMN14146978	SRR11247399	JAAKJT000000000
*Aeromonas caviae*	A702428	702428	Pakistan	Case	5,909,342	892,310,642	202.2	50	225,612	4,413,674	61.93	1,596	SAMN14146980	SRR11247397	JAAKJR000000000
*Aeromonas caviae*	A702434	702434	Pakistan	Case	5,898,360	890,652,360	201.9	68	148,516	4,410,618	61.93	1,596	SAMN14146981	SRR11247396	JAAKJQ000000000
*Aeromonas caviae*	A702464	702464	Pakistan	Case	5,708,948	862,051,148	195.9	58	141,817	4,400,074	61.9	1,602	SAMN14146982	SRR11247395	JAAKJP000000000
*Aeromonas caviae*	A702496	702496	Pakistan	Case	7,225,366	1,091,030,266	247.1	41	256,025	4,415,076	61.94	1,596	SAMN14146985	SRR11247392	JAAKJM000000000
*Aeromonas caviae*	A702540	702540	Pakistan	Case	8,272,942	1,249,214,242	273.6	195	57,205	4,565,230	61.04	1,439	SAMN14146986	SRR11247391	JAAKJL000000000
*Aeromonas caviae*	A702552	702552	Pakistan	Case	9,120,174	1,377,146,274	314.1	68	123,508	4,384,440	61.95	1,600	SAMN14146987	SRR11247389	JAAKJK000000000
*Aeromonas caviae*	A702590	702590	Pakistan	Case	7,001,846	1,057,278,746	239.0	73	153,139	4,423,633	61.92	1,596	SAMN14146988	SRR11247388	JAAKJJ000000000
*Aeromonas caviae*	A702615	702615	Pakistan	Case	8,991,880	1,357,773,880	309.8	69	120,625	4,382,727	61.97	1,603	SAMN14146990	SRR11247386	JAAKJH000000000
*Aeromonas caviae*	A702658	702658	Pakistan	Case	7,695,634	1,162,040,734	261.4	83	127,526	4,445,510	61.5	1,571	SAMN14146991	SRR11247385	JAAKJG000000000
*Aeromonas caviae*	A702699	702699	Pakistan	Case	4,474,914	675,712,014	151.1	64	147,585	4,472,195	61.94	1,477	SAMN14146992	SRR11247384	JAAKJF000000000
*Aeromonas caviae*	A702711	702711	Pakistan	Case	7,518,998	1,135,368,698	262.4	47	206,122	4,327,479	61.91	935	SAMN14146993	SRR11247383	JAAKJE000000000
*Aeromonas caviae*	A702831	702831	Pakistan	Case	8,029,014	1,212,381,114	275.3	338	24,998	4,403,461	61.64	313	SAMN14146996	SRR11247380	JAAKJB000000000
*Aeromonas caviae*	A702871	702871	Pakistan	Case	8,169,428	1,233,583,628	289.3	96	1,11,716	4,263,373	61.82	1,454	SAMN14146999	SRR11247376	JAAKIY000000000
*Aeromonas caviae*	A702903	702903	Pakistan	Case	10,139,240	1,531,025,240	349.4	47	220,048	4,382,308	61.93	1,604	SAMN14147000	SRR11247375	JAAKIX000000000
*Aeromonas caviae*	A702925	702925	Pakistan	Case	8,072,944	1,219,014,544	281.3	37	265,863	4,333,383	62.08	1,606	SAMN14147001	SRR11247374	JAAKIW000000000
*Aeromonas caviae*	A703054	703054	Pakistan	Case	7,013,126	1,058,982,026	234.0	337	24,977	4,525,007	61.24	1,440	SAMN14147005	SRR11247370	JAAKIS000000000
*Aeromonas caviae*	A703076	703076	Pakistan	Case	7,498,162	1,132,222,462	246.5	144	73,039	4,592,780	61.14	1,527	SAMN14147006	SRR11247369	JAAKIR000000000
*Aeromonas caviae*	A703089	703089	Pakistan	Case	6,900,000	1,041,900,000	239.1	85	148,927	4,357,304	61.55	1,462	SAMN14147007	SRR11247367	JAAKIQ000000000
*Aeromonas caviae*	A703097	703097	Pakistan	Case	7,125,558	1,075,959,258	251.0	78	121,439	4,287,453	61.75	1,451	SAMN14147008	SRR11247366	JAAKIP000000000
*Aeromonas caviae*	A703129	703129	Pakistan	Case	7,209,294	1,088,603,394	245.3	145	83,513	4,437,567	61.53	1,455	SAMN14147009	SRR11247365	JAAKIO000000000
*Aeromonas caviae*	A703164	703164	Pakistan	Case	6,909,758	1,043,373,458	231.2	179	79,547	4,513,200	61.15	1,499	SAMN14147011	SRR11247363	JAAKIM000000000
*Aeromonas caviae*	A703210	703210	Pakistan	Case	7,265,956	1,097,159,356	251.8	248	34,724	4,357,853	61.56	1,452	SAMN14147012	SRR11247362	JAAKIL000000000
*Aeromonas caviae*	A703254	703254	Pakistan	Case	7,826,358	1,181,780,058	264.5	133	72,587	4,467,282	61.39	1,442	SAMN14147016	SRR11247358	JAAKIH000000000
*Aeromonas caviae*	A703275	703275	Pakistan	Case	7,488,296	1,130,732,696	259.5	80	154,181	4,357,516	61.55	1,462	SAMN14147018	SRR11247355	JAAKIF000000000
*Aeromonas caviae*	A703340	703340	Pakistan	Case	7,296,120	1,101,714,120	253.8	52	200,594	4,341,465	62.07	1,549	SAMN14147022	SRR11247351	JAAKIB000000000
*Aeromonas caviae*	A703372	703372	Pakistan	Case	7,400,540	1,117,481,540	253.5	57	167,321	4,407,709	61.73	1,507	SAMN14147024	SRR11247349	JAAKHZ000000000
*Aeromonas caviae*	A703439	703439	Pakistan	Case	8,276,412	1,249,738,212	278.0	96	93,658	4,494,909	61.68	1,605	SAMN14147026	SRR11247347	JAAKHX000000000
*Aeromonas caviae*	A703548	703548	Pakistan	Case	7,442,020	1,123,745,020	257.2	51	175,654	4,369,939	61.98	1,609	SAMN14147031	SRR11247341	JAAKHS000000000
*Aeromonas caviae*	A703603	703603	Pakistan	Case	7,803,190	1,178,281,690	264.6	63	163,441	4,453,393	61.57	1,553	SAMN14147033	SRR11247339	JAAKHQ000000000
*Aeromonas caviae*	A703667	703667	Pakistan	Case	5,904,428	891,568,628	201.1	232	46,928	4,433,685	61.31	1,528	SAMN14147037	SRR11247334	JAAKHM000000000
*Aeromonas caviae*	A703724	703724	Pakistan	Case	3,840,012	579,841,812	133.9	89	106,961	4,331,273	61.79	1,516	SAMN14147077	SRR11247289	JAAKWO000000000
*Aeromonas caviae*	A703819	703819	Pakistan	Case	5,933,614	895,975,714	208.2	116	75,248	4,303,294	61.76	1,490	SAMN14147039	SRR11247332	JAAKHK000000000
*Aeromonas caviae*	A703853	703853	Pakistan	Case	6,900,144	1,041,921,744	243.5	100	114,664	4,279,143	61.78	1,252	SAMN14147040	SRR11247331	JAAKHJ000000000
*Aeromonas caviae*	A703914	703914	Pakistan	Case	7,407,142	1,118,478,442	249.5	54	139,427	4,482,013	61.6	1,610	SAMN14147042	SRR11247329	JAAKHH000000000
*Aeromonas caviae*	A704013	704013	Pakistan	Case	6,789,756	1,025,253,156	229.5	188	44,550	4,467,423	61.41	1,446	SAMN14147044	SRR11247327	JAAKHF000000000
*Aeromonas caviae*	A704081	704081	Pakistan	Case	6,451,370	974,156,870	214.4	131	80,486	4,544,453	61.22	1,615	SAMN14147048	SRR11247321	JAAKXR000000000
*Aeromonas caviae*	A704099	704099	Pakistan	Case	8,562,856	1,292,991,256	300.0	91	97,252	4,309,735	61.81	509	SAMN14147049	SRR11247320	JAAKXQ000000000
*Aeromonas caviae*	A704158	704158	Pakistan	Case	6,818,220	1,029,551,220	240.3	40	177,154	4,283,934	61.89	1,617	SAMN14147052	SRR11247317	JAAKXN000000000
*Aeromonas caviae*	A704205	704205	Pakistan	Case	7,842,448	1,184,209,648	256.8	164	69,640	4,611,583	61.2	1,459	SAMN14147053	SRR11247316	JAAKXM000000000
*Aeromonas caviae*	A704263	704263	Pakistan	Case	6,681,464	1,008,901,064	225.4	182	49,943	4,476,101	61.24	1,445	SAMN14147055	SRR11247314	JAAKXK000000000
*Aeromonas caviae*	A500116	500116	India	Control	6,844,932	1,033,584,732	218.4	79	118,982	4,733,196	60.28	1,520	SAMN14146647	SRR11247486	JAAKWM000000000
*Aeromonas caviae*	A503742	503742	India	Control	6,561,406	990,772,306	224.9	92	96,562	4,404,916	61.41	1,466	SAMN14146652	SRR11247281	JAAKWH000000000
*Aeromonas caviae*	A600105	600105	Bangladesh	Control	6,575,738	992,936,438	221.9	164	55,132	4,473,908	61.26	313	SAMN14146654	SRR11247259	JAAKWF000000000
*Aeromonas caviae*	A600590	600590	Bangladesh	Control	9,009,654	1,360,457,754	313.6	106	103,316	4,337,623	61.48	1,546	SAMN14146693	SRR11247285	JAAKUS000000000
*Aeromonas caviae*	A600812	600812	Bangladesh	Control	7,468,460	1,127,737,460	259.9	44	176,952	4,338,342	61.92	1,456	SAMN14146706	SRR11247271	JAAKUF000000000
*Aeromonas caviae*	A601023	601023	Bangladesh	Control	6,955,374	1,050,261,474	243.5	50	163,890	4,312,324	61.82	1,552	SAMN14146723	SRR11247252	JAAKTO000000000
*Aeromonas caviae*	A602416	602416	Bangladesh	Control	6,158,004	929,858,604	211.6	148	76,859	4,394,105	61.43	1,259	SAMN14146761	SRR11247468	JAAKSC000000000
*Aeromonas caviae*	A602633	602633	Bangladesh	Control	5,288,432	798,553,232	181.3	100	96,888	4,405,390	61.59	1,458	SAMN14146782	SRR11247447	JAAKRH000000000
*Aeromonas caviae*	A602757	602757	Bangladesh	Control	6,024,166	909,649,066	207.6	37	252,626	4,382,382	62.01	1,566	SAMN14146789	SRR11247439	JAAKRA000000000
*Aeromonas caviae*	A603002	603002	Bangladesh	Control	9,160,788	1,383,278,988	307.1	168	70,402	4,504,366	61.27	1,351	SAMN14146811	SRR11247243	JAAKQE000000000
*Aeromonas caviae*	A603316	603316	Bangladesh	Control	8,339,650	1,259,287,150	291.8	46	185,295	4,315,719	62.04	1,483	SAMN14146830	SRR11247222	JAAKPL000000000
*Aeromonas caviae*	A603539	603539	Bangladesh	Control	6,525,814	985,397,914	221.2	162	55,448	4,454,253	61.38	1,479	SAMN14146839	SRR11247212	JAAKPC000000000
*Aeromonas caviae*	A603709	603709	Bangladesh	Control	7,150,312	1,079,697,112	246.7	40	253,734	4,375,948	61.98	1,577	SAMN14146858	SRR11247190	JAAKOJ000000000
*Aeromonas caviae*	A603746	603746	Bangladesh	Control	7,008,088	1,058,221,288	247.8	61	151,775	4,270,724	61.98	1,467	SAMN14146860	SRR11247188	JAAKOH000000000
*Aeromonas caviae*	A603917	603917	Bangladesh	Control	8,373,874	1264,454,974	290.1	39	193,080	4,357,937	61.82	1,554	SAMN14146870	SRR11247177	JAAKNX000000000
*Aeromonas caviae*	A604018	604018	Bangladesh	Control	6,360,348	960,412,548	215.1	141	67,153	4,463,938	61.5	1,511	SAMN14146877	SRR11247167	JAAKNQ000000000
*Aeromonas caviae*	A604270	604270	Bangladesh	Control	5,874,762	887,089,062	199.3	41	224,619	4,450,118	61.91	1,583	SAMN14146894	SRR11247149	JAAKMZ000000000
*Aeromonas caviae*	A604329	604329	Bangladesh	Control	6,554,514	989,731,614	223.3	62	147,130	4,431,707	61.62	1,503	SAMN14146900	SRR11247142	JAAKMT000000000
*Aeromonas caviae*	A604381	604381	Bangladesh	Control	8,085,870	1,220,966,370	271.0	70	119,508	4,504,642	61.55	1,585	SAMN14146905	SRR11247137	JAAKMO000000000
*Aeromonas caviae*	A604469	604469	Bangladesh	Control	8,357,844	1,262,034,444	282.1	142	67,791	4,473,839	61.34	1,587	SAMN14146914	SRR11247127	JAAKMF000000000
*Aeromonas caviae*	A604542	604542	Bangladesh	Control	6,590,808	995,212,008	223.2	168	57,123	4,458,679	61.41	1,328	SAMN14146923	SRR11247117	JAAKLW000000000
*Aeromonas caviae*	A700078	700078	Pakistan	Control	6,591,644	995,338,244	229.9	94	102,280	4,328,670	61.78	1,590	SAMN14146932	SRR11247107	JAAKLN000000000
*Aeromonas caviae*	A700302	700302	Pakistan	Control	9,945,504	1,501,771,104	340.7	169	61,392	4,407,633	61.38	1,593	SAMN14146939	SRR11247099	JAAKLG000000000
*Aeromonas caviae*	A700355	700355	Pakistan	Control	6,706,278	1,012,647,978	238.7	68	123,761	4,243,018	61.88	1,464	SAMN14146944	SRR11247094	JAAKLB000000000
*Aeromonas caviae*	A700555	700555	Pakistan	Control	7,853,464	1,185,873,064	265.6	80	132,734	4,465,437	61.34	1,485	SAMN14146954	SRR11247082	JAAKKR000000000
*Aeromonas caviae*	A700991	700991	Pakistan	Control	8,400,010	1,268,401,510	300.2	98	104,106	4,224,626	61.93	1,595	SAMN14146966	SRR11247413	JAAKKF000000000
*Aeromonas caviae*	A702427	702427	Pakistan	Control	7,073,034	1,068,028,134	241.6	105	86,870	4,421,419	61.83	1,602	SAMN14146979	SRR11247398	JAAKJS000000000
*Aeromonas caviae*	A702465	702465	Pakistan	Control	6,763,952	1,021,356,752	231.3	42	234,446	4,415,024	61.94	1,596	SAMN14146983	SRR11247394	JAAKJO000000000
*Aeromonas caviae*	A702469	702469	Pakistan	Control	7,466,678	1,127,468,378	255.2	41	234,446	4,417,613	61.94	1,596	SAMN14146984	SRR11247393	JAAKJN000000000
*Aeromonas caviae*	A702606	702606	Pakistan	Control	4,824,158	728,447,858	152.9	192	70,093	4,764,718	61.19	1,602	SAMN14146989	SRR11247387	JAAKJI000000000
*Aeromonas caviae*	A703040	703040	Pakistan	Control	6,816,832	1,029,341,632	233.3	176	67,515	4,412,993	61.53	1,478	SAMN14147003	SRR11247372	JAAKIU000000000
*Aeromonas caviae*	A703046	703046	Pakistan	Control	10,545,072	1,592,305,872	347.8	168	58,184	4,578,688	61.1	1,442	SAMN14147004	SRR11247371	JAAKIT000000000
*Aeromonas caviae*	A703142	703142	Pakistan	Control	6,031,288	910,724,488	207.7	92	116,323	4,385,124	62.03	1,607	SAMN14147010	SRR11247364	JAAKIN000000000
*Aeromonas caviae*	A703236	703236	Pakistan	Control	5,061,346	764,263,246	172.5	129	80,450	4,431,726	61.52	313	SAMN14147014	SRR11247360	JAAKIJ000000000
*Aeromonas caviae*	A703317	703317	Pakistan	Control	7,483,398	1,129,993,098	257.8	60	126,367	4,384,016	61.98	1,608	SAMN14147020	SRR11247353	JAAKID000000000
*Aeromonas caviae*	A703472	703472	Pakistan	Control	6,715,546	1,014,047,446	227.5	192	54,705	4,456,734	61.3	1,508	SAMN14147027	SRR11247345	JAAKHW000000000
*Aeromonas caviae*	A703524	703524	Pakistan	Control	7,687,612	1,160,829,412	261.6	160	59,303	4,436,630	61.22	1,534	SAMN14147029	SRR11247343	JAAKHU000000000
*Aeromonas caviae*	A703530	703530	Pakistan	Control	5,210,304	786,755,904	179.8	120	84,115	4,375,587	61.76	1,252	SAMN14147030	SRR11247342	JAAKHT000000000
*Aeromonas caviae*	A703620	703620	Pakistan	Control	7,145,036	1,078,900,436	244.1	118	86,218	4,420,670	61.29	1,531	SAMN14147034	SRR11247338	JAAKHP000000000
*Aeromonas caviae*	A703636	703636	Pakistan	Control	6,434,760	971,648,760	220.0	184	52,340	4,417,411	61.83	1,599	SAMN14147036	SRR11247336	JAAKHN000000000
*Aeromonas caviae*	A704116	704116	Pakistan	Control	6,306,526	952,285,426	211.1	60	174,632	4,510,640	61.27	1,616	SAMN14147050	SRR11247319	JAAKXP000000000
*Aeromonas dhakensis*	A600276	600276	Bangladesh	Case	3,769,296	569,163,696	122.4	47	215,762	4,651,209	61.95	308	SAMN14146665	SRR11247226	JAAKVU000000000
*Aeromonas dhakensis*	A602444	602444	Bangladesh	Case	7,811,652	1,179,559,452	251.8	60	165,096	4,684,050	61.8	1,623	SAMN14146767	SRR11247461	JAAKRW000000000
*Aeromonas dhakensis*	A603084	603084	Bangladesh	Case	8,762,998	1,323,212,698	277.6	50	241,000	4,767,047	61.64	1,624	SAMN14146819	SRR11247234	JAAKPW000000000
*Aeromonas dhakensis*	A603314	603314	Bangladesh	Case	7,661,238	1,156,846,938	247.1	40	289,325	4,681,277	61.89	1,621	SAMN14146829	SRR11247223	JAAKPM000000000
*Aeromonas dhakensis*	A603610	603610	Bangladesh	Case	7,665,594	1,157,504,694	250.4	37	288,726	4,622,988	61.94	1,622	SAMN14146848	SRR11247201	JAAKOT000000000
*Aeromonas dhakensis*	A604199	604199	Bangladesh	Case	7,546,760	1,139,560,760	233.8	139	54,676	4,873,238	61.52	1,632	SAMN14146892	SRR11247151	JAAKNB000000000
*Aeromonas dhakensis*	A700052	700052	Pakistan	Case	3,736,180	564,163,180	116.4	39	236,962	4,846,684	61.58	1,630	SAMN14146929	SRR11247110	JAAKLQ000000000
*Aeromonas dhakensis*	A700388	700388	Pakistan	Case	10,508,046	1,586,714,946	331.3	61	144,090	4,789,405	61.79	1,633	SAMN14146947	SRR11247089	JAAKKY000000000
*Aeromonas dhakensis*	A700482	700482	Pakistan	Case	8,408,710	1,269,715,210	264.3	66	139,769	4,804,833	61.77	1,634	SAMN14146950	SRR11247086	JAAKKV000000000
*Aeromonas dhakensis*	A700546	700546	Pakistan	Case	5,915,210	893,196,710	184.6	42	269,974	4,839,220	61.4	1,631	SAMN14146952	SRR11247084	JAAKKT000000000
*Aeromonas dhakensis*	A700694	700694	Pakistan	Case	9,106,846	1,375,133,746	290.4	129	83,681	4,735,565	61.79	1,626	SAMN14146957	SRR11247422	JAAKKO000000000
*Aeromonas dhakensis*	A700812	700812	Pakistan	Case	7,533,030	1,137,487,530	236.6	284	30,752	4,807,319	61.73	ND	SAMN14146964	SRR11247415	JAAKKH000000000
*Aeromonas dhakensis*	A702794	702794	Pakistan	Case	8,193,320	1,237,191,320	257.4	127	68,355	4,805,774	61.73	1,620	SAMN14146995	SRR11247381	JAAKJC000000000
*Aeromonas dhakensis*	A703272	703272	Pakistan	Case	7,819,132	1,180,688,932	247.5	247	38,616	4,770,224	61.71	ND	SAMN14147017	SRR11247356	JAAKIG000000000
*Aeromonas dhakensis*	A703287	703287	Pakistan	Case	7,498,428	1,132,262,628	233.0	79	144,077	4,859,420	61.47	1,629	SAMN14147019	SRR11247354	JAAKIE000000000
*Aeromonas dhakensis*	A703364	703364	Pakistan	Case	7,187,622	1,085,330,922	226.0	45	167,956	4,802,152	61.5	1,635	SAMN14147023	SRR11247350	JAAKIA000000000
*Aeromonas dhakensis*	A703754	703754	Pakistan	Case	8,712,954	1,315,656,054	285.0	39	263,442	4,616,779	61.95	1,628	SAMN14147038	SRR11247333	JAAKHL000000000
*Aeromonas dhakensis*	A600752	600752	Bangladesh	Control	9,339,854	1,410,317,954	293.0	48	215,317	4,813,267	61.52	1,619	SAMN14146701	SRR11247276	JAAKUK000000000
*Aeromonas dhakensis*	A700698	700698	Pakistan	Control	9,702,110	1,465,018,610	315.1	145	65,286	4,649,244	61.96	1,627	SAMN14146958	SRR11247421	JAAKKN000000000
*Aeromonas dhakensis*	A704020	704020	Pakistan	Control	6,750,498	1,019,325,198	217.7	39	236,145	4,682,998	61.98	1,625	SAMN14147045	SRR11247326	JAAKXU000000000
*Aeromonas dhakensis*	A704258	704258	Pakistan	Control	6,181,832	933,456,632	196.9	35	222,114	4,741,214	61.97	1,618	SAMN14147054	SRR11247315	JAAKXL000000000
*Aeromonas enteropelogenes*	A600259	600259	Bangladesh	Case	7,425,706	1,121,281,606	252.9	89	105,150	4,434,329	59.82	1,641	SAMN14146664	SRR11247237	JAAKVV000000000
*Aeromonas enteropelogenes*	A600280	600280	Bangladesh	Case	3,347,620	505,490,620	113.8	38	277,020	4,443,018	59.84	1,642	SAMN14146667	SRR11247203	JAAKVS000000000
*Aeromonas enteropelogenes*	A600625	600625	Bangladesh	Case	7,847,286	1,184,940,186	252.3	53	235,356	4,697,311	59.43	1,645	SAMN14146695	SRR11247283	JAAKUQ000000000
*Aeromonas enteropelogenes*	A600847	600847	Bangladesh	Case	3,781,148	570,953,348	126.8	54	170,173	4,501,836	59.78	1,659	SAMN14147069	SRR11247298	JAAKWW000000000
*Aeromonas enteropelogenes*	A601057	601057	Bangladesh	Case	3,809,496	575,233,896	127.7	31	310,234	4,503,834	59.67	1,661	SAMN14147072	SRR11247295	JAAKWT000000000
*Aeromonas enteropelogenes*	A601059	601059	Bangladesh	Case	3,335,812	503,707,612	116.2	47	207,670	4,336,373	60.02	1,647	SAMN14146728	SRR11247506	JAAKTJ000000000
*Aeromonas enteropelogenes*	A601243	601243	Bangladesh	Case	4,172,856	630,101,256	143.6	47	189,190	4,386,858	59.93	1,638	SAMN14146744	SRR11247489	JAAKST000000000
*Aeromonas enteropelogenes*	A602215	602215	Bangladesh	Case	8,494,870	1,282,725,370	287.3	34	244,627	4,464,587	59.83	1,649	SAMN14146753	SRR11247477	JAAKSK000000000
*Aeromonas enteropelogenes*	A603057	603057	Bangladesh	Case	5,843,870	882,424,370	200.1	107	81,994	4,410,344	59.67	1,636	SAMN14146817	SRR11247236	JAAKPY000000000
*Aeromonas enteropelogenes*	A603975	603975	Bangladesh	Case	7,296,950	1,101,839,450	239.6	41	307,577	4,597,988	59.72	1,654	SAMN14146873	SRR11247174	JAAKNU000000000
*Aeromonas enteropelogenes*	A604086	604086	Bangladesh	Case	7,959,014	1,201,811,114	258.9	31	307,460	4,641,597	59.65	1,654	SAMN14146883	SRR11247161	JAAKNK000000000
*Aeromonas enteropelogenes*	A604446	604446	Bangladesh	Case	7,216,474	1,089,687,574	233.4	40	271,799	4,668,678	59.58	1,655	SAMN14146912	SRR11247129	JAAKMH000000000
*Aeromonas enteropelogenes*	A700320	700320	Pakistan	Case	5,899,888	890,883,088	199.7	187	48,494	4,462,224	59.6	1,636	SAMN14146940	SRR11247098	JAAKLF000000000
*Aeromonas enteropelogenes*	A700809	700809	Pakistan	Case	8,092,600	1,221,982,600	280.6	43	205,188	4,354,314	60.04	1,637	SAMN14146963	SRR11247416	JAAKKI000000000
*Aeromonas enteropelogenes*	A702123	702123	Pakistan	Case	7,635,044	1,152,891,644	243.8	75	142,879	4,728,616	59.08	1,638	SAMN14146970	SRR11247408	JAAKKB000000000
*Aeromonas enteropelogenes*	A702870	702870	Pakistan	Case	10,472,270	1,581,312,770	364.5	40	266,803	4,338,689	59.9	1,657	SAMN14146998	SRR11247377	JAAKIZ000000000
*Aeromonas enteropelogenes*	A703569	703569	Pakistan	Case	8,057,770	1,216,723,270	265.6	87	113,979	4,580,265	59.75	1,658	SAMN14147032	SRR11247340	JAAKHR000000000
*Aeromonas enteropelogenes*	A703632	703632	Pakistan	Case	6,790,776	1,025,407,176	227.9	77	200,396	4,499,099	59.55	1,640	SAMN14147035	SRR11247337	JAAKHO000000000
*Aeromonas enteropelogenes*	A600445	600445	Bangladesh	Control	7,511,638	1,134,257,338	249.8	26	305,382	4,540,901	59.78	1,643	SAMN14146678	SRR11247079	JAAKVH000000000
*Aeromonas enteropelogenes*	A600616	600616	Bangladesh	Control	6,563,800	991,133,800	220.7	38	316,929	4,490,482	59.81	1,644	SAMN14146694	SRR11247284	JAAKUR000000000
*Aeromonas enteropelogenes*	A600777	600777	Bangladesh	Control	6,823,280	1,030,315,280	218.5	60	248,318	4,715,543	59.44	1,639	SAMN14146703	SRR11247274	JAAKUI000000000
*Aeromonas enteropelogenes*	A600966	600966	Bangladesh	Control	7,380,022	1,114,383,322	237.1	71	147,428	4,700,223	59.5	1,646	SAMN14146717	SRR11247258	JAAKTU000000000
*Aeromonas enteropelogenes*	A600982	600982	Bangladesh	Control	4,122,626	622,516,526	143.2	38	209,608	4,346,967	60.01	1,660	SAMN14147071	SRR11247296	JAAKWU000000000
*Aeromonas enteropelogenes*	A601093	601093	Bangladesh	Control	6,768,880	1,022,100,880	227.2	36	213,940	4,499,254	59.75	1,648	SAMN14146733	SRR11247501	JAAKTE000000000
*Aeromonas enteropelogenes*	A602357	602357	Bangladesh	Control	9,466,656	1,429,465,056	319.4	49	195,595	4,474,881	59.84	1,650	SAMN14146759	SRR11247470	JAAKSE000000000
*Aeromonas enteropelogenes*	A602887	602887	Bangladesh	Control	7,551,736	1,140,312,136	261.3	35	230,251	4,364,596	59.61	1,651	SAMN14146801	SRR11247426	JAAKQO000000000
*Aeromonas enteropelogenes*	A603203	603203	Bangladesh	Control	7,495,612	1,131,837,412	248.0	38	284,011	4,563,636	59.77	1,656	SAMN14146823	SRR11247230	JAAKPS000000000
*Aeromonas enteropelogenes*	A603682	603682	Bangladesh	Control	5,409,688	816,862,888	188.3	81	123,898	4,337,929	59.77	1,652	SAMN14146854	SRR11247195	JAAKON000000000
*Aeromonas enteropelogenes*	A603753	603753	Bangladesh	Control	3,277,250	494,864,750	109.9	50	192,076	4,501,777	59.73	1,653	SAMN14146862	SRR11247186	JAAKOF000000000
*Aeromonas hydrophila*	A603261	603261	Bangladesh	Case	7,559,198	1,141,438,898	244.3	38	361,278	4,672,022	61.41	1,662	SAMN14146827	SRR11247225	JAAKPO000000000
*Aeromonas jandaei*	A503480	503480	India	Case	7,670,186	1,158,198,086	255.5	29	250,835	4,533,439	59.09	1,664	SAMN14146651	SRR11247324	JAAKWI000000000
*Aeromonas jandaei*	A600576	600576	Bangladesh	Case	5,873,302	886,868,602	205.2	32	212,022	4,321,368	59.16	1,665	SAMN14146691	SRR11247287	JAAKUU000000000
*Aeromonas jandaei*	A600974	600974	Bangladesh	Case	7,396,908	1,116,933,108	250.8	66	129,979	4,452,781	59.07	1,668	SAMN14146719	SRR11247256	JAAKTS000000000
*Aeromonas jandaei*	A602766	602766	Bangladesh	Case	5,196,308	784,642,508	174.6	51	212,159	4,492,776	58.93	1,670	SAMN14146795	SRR11247433	JAAKQU000000000
*Aeromonas jandaei*	A602936	602936	Bangladesh	Case	7,303,900	1,102,888,900	238.6	45	185,433	4,621,750	58.67	1,671	SAMN14146807	SRR11247247	JAAKQI000000000
*Aeromonas jandaei*	A603229	603229	Bangladesh	Case	5,171,048	780,828,248	176.5	37	239,515	4,424,916	59.19	1,673	SAMN14146824	SRR11247229	JAAKPR000000000
*Aeromonas jandaei*	A604369	604369	Bangladesh	Case	7,422,060	1,120,731,060	241.4	255	41,448	4,643,260	58.74	1,672	SAMN14146903	SRR11247139	JAAKMQ000000000
*Aeromonas jandaei*	A604402	604402	Bangladesh	Case	8,802,860	1,329,231,860	297.6	61	126,938	4,467,195	59.1	1,674	SAMN14146906	SRR11247136	JAAKMN000000000
*Aeromonas jandaei*	A604434	604434	Bangladesh	Case	7,898,736	1,192,709,136	267.5	149	66,024	4,458,409	59	1,675	SAMN14146910	SRR11247131	JAAKMJ000000000
*Aeromonas jandaei*	A604487	604487	Bangladesh	Case	7,345,912	1,109,232,712	248.7	45	249,667	4,460,366	58.98	1,676	SAMN14146917	SRR11247123	JAAKMC000000000
*Aeromonas jandaei*	A600694	600694	Bangladesh	Control	6,922,844	1,045,349,444	226.1	50	190,498	4,623,714	58.7	1,666	SAMN14146696	SRR11247282	JAAKUP000000000
*Aeromonas jandaei*	A600964	600964	Bangladesh	Control	5,993,412	905,005,212	208.7	35	229,774	4,337,075	58.84	1,667	SAMN14146715	SRR11247261	JAAKTW000000000
*Aeromonas jandaei*	A601150	601150	Bangladesh	Control	7,540,942	1,138,682,242	262.1	36	191,418	4,343,833	59.19	1,669	SAMN14146740	SRR11247493	JAAKSX000000000
*Aeromonas jandaei*	A703249	703249	Pakistan	Control	5,556,142	838,977,442	189.0	48	213,263	4,438,028	59.05	1,663	SAMN14147015	SRR11247359	JAAKII000000000
*Aeromonas sanarellii*	A600351	600351	Bangladesh	Case	7,202,114	1,087,519,214	258.7	43	223,586	4,204,165	63.04	ND	SAMN14146673	SRR11247135	JAAKVM000000000
*Aeromonas sanarellii*	A703232	703232	Pakistan	Case	5,540,364	836,594,964	194.6	67	118,180	4,298,310	63	1,678	SAMN14147013	SRR11247361	JAAKIK000000000
*Aeromonas sanarellii*	A602534	602534	Bangladesh	Control	5,790,248	874,327,448	203.6	105	76,666	4,294,518	63.03	1,677	SAMN14146775	SRR11247455	JAAKRO000000000
*Aeromonas sanarellii*	A604417	604417	Bangladesh	Control	7,368,622	1,112,661,922	264.7	49	215,891	4,202,960	63.03	ND	SAMN14146907	SRR11247134	JAAKMM000000000
*Aeromonas taiwanensis*	A601142	601142	Bangladesh	Case	4,188,870	632,519,370	141.7	141	70,391	4,463,448	62.06	1,715	SAMN14147073	SRR11247294	JAAKWS000000000
*Aeromonas taiwanensis*	A602536	602536	Bangladesh	Case	6,130,482	925,702,782	216.8	58	141,414	4,269,926	62.39	1,714	SAMN14146777	SRR11247452	JAAKRM000000000
*Aeromonas taiwanensis*	A603101	603101	Bangladesh	Case	5,908,992	892,257,792	204.4	95	137,530	4,365,042	62.22	1,710	SAMN14146820	SRR11247233	JAAKPV000000000
*Aeromonas taiwanensis*	A600450	600450	Bangladesh	Control	6,482,022	978,785,322	222.1	105	117,912	4,406,134	62.23	1,710	SAMN14146679	SRR11247412	JAAKVG000000000
*Aeromonas taiwanensis*	A601195	601195	Bangladesh	Control	6,067,288	916,160,488	210.8	50	172,121	4,345,261	62.39	1,713	SAMN14146742	SRR11247491	JAAKSV000000000
*Aeromonas taiwanensis*	A601281	601281	Bangladesh	Control	7,526,854	1,136,554,954	260.9	104	118,461	4,356,162	62.24	1,710	SAMN14146746	SRR11247487	JAAKSR000000000
*Aeromonas taiwanensis*	A601303	601303	Bangladesh	Control	5,862,596	885,251,996	207.6	43	173,706	4,264,715	62.39	1,714	SAMN14146748	SRR11247482	JAAKSP000000000
*Aeromonas taiwanensis*	A602758	602758	Bangladesh	Control	7,383,420	1,114,896,420	256.3	68	134,890	4,349,880	62.59	1,709	SAMN14146790	SRR11247438	JAAKQZ000000000
*Aeromonas veronii*	A600479	600479	Bangladesh	Case	8,004,044	1,208,610,644	265.0	39	164,725	4,561,025	58.48	1,695	SAMN14146680	SRR11247401	JAAKVF000000000
*Aeromonas veronii*	A600556	600556	Bangladesh	Case	3,458,224	522,191,824	115.4	61	125,130	4,524,668	58.42	1,691	SAMN14147062	SRR11247306	JAAKXD000000000
*Aeromonas veronii*	A600620	600620	Bangladesh	Case	4,675,904	706,061,504	158.5	36	254,235	4,455,491	58.83	1,708	SAMN14147064	SRR11247304	JAAKXB000000000
*Aeromonas veronii*	A600724	600724	Bangladesh	Case	4,376,804	660,897,404	144.4	44	182,092	4,575,408	58.47	1,687	SAMN14146699	SRR11247278	JAAKUM000000000
*Aeromonas veronii*	A600774	600774	Bangladesh	Case	7,437,142	1,123,008,442	252.9	38	225,095	4,439,896	58.65	1,696	SAMN14146702	SRR11247275	JAAKUJ000000000
*Aeromonas veronii*	A600948	600948	Bangladesh	Case	6,905,552	1,042,738,352	236.5	52	156,467	4,408,817	58.73	1,688	SAMN14146713	SRR11247263	JAAKTY000000000
*Aeromonas veronii*	A601027	601027	Bangladesh	Case	3,654,438	551,820,138	117.0	37	258,610	4,717,485	58.42	1,689	SAMN14146724	SRR11247251	JAAKTN000000000
*Aeromonas veronii*	A601039	601039	Bangladesh	Case	4,954,552	748,137,352	169.1	55	153,193	4,424,902	58.72	1,698	SAMN14146726	SRR11247509	JAAKTL000000000
*Aeromonas veronii*	A601115	601115	Bangladesh	Case	6,439,286	972,332,186	220.3	46	155,440	4,414,001	58.7	1,690	SAMN14146734	SRR11247500	JAAKTD000000000
*Aeromonas veronii*	A602293	602293	Bangladesh	Case	6,610,102	998,125,402	225.0	66	124,620	4,436,712	58.89	1,699	SAMN14146756	SRR11247474	JAAKSH000000000
*Aeromonas veronii*	A602658	602658	Bangladesh	Case	8,220,204	1,241,250,804	278.9	72	116,909	4,450,270	58.79	1,701	SAMN14146784	SRR11247445	JAAKRF000000000
*Aeromonas veronii*	A602826	602826	Bangladesh	Case	5,998,498	905,773,198	200.3	51	166,437	4,522,603	58.6	1,692	SAMN14146799	SRR11247428	JAAKQQ000000000
*Aeromonas veronii*	A603079	603079	Bangladesh	Case	6,135,646	926,482,546	204.5	54	155,332	4,530,479	58.46	1,705	SAMN14146818	SRR11247235	JAAKPX000000000
*Aeromonas veronii*	A603359	603359	Bangladesh	Case	6,990,196	1,055,519,596	235.4	44	187,185	4,484,647	58.65	1,693	SAMN14146831	SRR11247221	JAAKPK000000000
*Aeromonas veronii*	A603404	603404	Bangladesh	Case	8,175,188	1,234,453,388	277.5	31	291,568	4,448,586	58.91	1,702	SAMN14146837	SRR11247214	JAAKPE000000000
*Aeromonas veronii*	A603607	603607	Bangladesh	Case	6,804,312	1,027,451,112	228.0	44	171,378	4,507,114	58.65	1,684	SAMN14146846	SRR11247205	JAAKOV000000000
*Aeromonas veronii*	A603696	603696	Bangladesh	Case	6,605,402	997,415,702	213.6	99	125,102	4,668,667	58.41	1,694	SAMN14146857	SRR11247191	JAAKOK000000000
*Aeromonas veronii*	A603757	603757	Bangladesh	Case	6,902,632	1,042,297,432	234.5	39	166,599	4,444,561	58.67	1,703	SAMN14146863	SRR11247185	JAAKOE000000000
*Aeromonas veronii*	A604015	604015	Bangladesh	Case	6,926,556	1,045,909,956	234.7	58	165,105	4,456,280	58.64	1,686	SAMN14146876	SRR11247171	JAAKNR000000000
*Aeromonas veronii*	A604176	604176	Bangladesh	Case	3,683,212	55,6165,012	122.9	320	29,106	4,524,657	58.75	1,680	SAMN14146889	SRR11247154	JAAKNE000000000
*Aeromonas veronii*	A604282	604282	Bangladesh	Case	7,305,658	1,103,154,358	248.9	35	229,485	4,432,568	58.62	1,707	SAMN14146898	SRR11247144	JAAKMV000000000
*Aeromonas veronii*	A604443	604443	Bangladesh	Case	6,027,068	910,087,268	202.8	34	292,206	4,487,197	58.7	1,685	SAMN14146911	SRR11247130	JAAKMI000000000
*Aeromonas veronii*	A604534	604534	Bangladesh	Case	7,821,060	1,180,980,060	250.9	59	154,013	4,707,308	58.31	966	SAMN14146922	SRR11247118	JAAKLX000000000
*Aeromonas veronii*	A700377	700377	Pakistan	Case	6,435,350	971,737,850	216.3	42	257,625	4,491,836	58.65	1,700	SAMN14146945	SRR11247093	JAAKLA000000000
*Aeromonas veronii*	A700722	700722	Pakistan	Case	7,720,242	1,165,756,542	255.3	300	32,479	4,565,909	58.47	1,704	SAMN14146960	SRR11247419	JAAKKL000000000
*Aeromonas veronii*	A500451	500451	India	Case	3,478,550	525,261,050	118.5	33	175,930	4,432,680	58.94	1,780	SAMN14146649	SRR11247204	JAAKWK000000000
*Aeromonas veronii*	A504091	504091	India	Case	7,748,796	1,170,068,196	255.6	71	147,453	4,576,955	58.68	1,737	SAMN14146653	SRR11247270	JAAKWG000000000
*Aeromonas veronii*	A600120	600120	Bangladesh	Case	3,457,388	522,065,588	116.7	43	221,317	4,474,168	58.84	1,747	SAMN14146655	SRR11247508	JAAKWE000000000
*Aeromonas veronii*	A600126	600126	Bangladesh	Case	3,790,796	572,410,196	128.2	32	364,331	4,465,527	58.77	1,719	SAMN14146656	SRR11247497	JAAKWD000000000
*Aeromonas veronii*	A600143	600143	Bangladesh	Case	4,682,120	702,318,000	175.1	163	47,568	4,011,918	58.84	ND	SAMN14146658	SRR11247473	JAAKWB000000000
*Aeromonas veronii*	A600174	600174	Bangladesh	Case	15,545,206	2,347,326,106	543.9	139	61,611	4,315,834	58.86	1,727	SAMN14147057	SRR11247311	JAAKXI000000000
*Aeromonas veronii*	A600189	600189	Bangladesh	Case	3,506,732	529,516,532	122.7	137	60,391	4,315,031	58.86	1,727	SAMN14146659	SRR11247462	JAAKWA000000000
*Aeromonas veronii*	A600193	600193	Bangladesh	Case	9,160,720	1,383,268,720	295.5	56	151,653	4,680,570	58.71	1,743	SAMN14146660	SRR11247453	JAAKVZ000000000
*Aeromonas veronii*	A600216	600216	Bangladesh	Case	4,469,370	674,874,870	151.0	46	199,115	4,470,417	58.76	1,744	SAMN14146662	SRR11247431	JAAKVX000000000
*Aeromonas veronii*	A600257	600257	Bangladesh	Case	3,683,196	556,162,596	124.9	43	205,152	4,451,814	58.73	1,779	SAMN14146663	SRR11247248	JAAKVW000000000
*Aeromonas veronii*	A600323	600323	Bangladesh	Case	6,690,506	1,010,266,406	227.3	32	206,575	4,443,722	58.88	1,745	SAMN14146670	SRR11247168	JAAKVP000000000
*Aeromonas veronii*	A600337	600337	Bangladesh	Case	6,978,356	1,053,731,756	236.7	31	211,264	4,451,041	58.81	1,746	SAMN14146672	SRR11247146	JAAKVN000000000
*Aeromonas veronii*	A600357	600357	Bangladesh	Case	3,271,278	493,962,978	110.6	52	192,316	4,468,098	58.86	1,750	SAMN14147060	SRR11247308	JAAKXF000000000
*Aeromonas veronii*	A600497	600497	Bangladesh	Case	4,273,290	645,266,790	143.7	51	191,069	4,489,822	58.65	1,748	SAMN14146681	SRR11247390	JAAKVE000000000
*Aeromonas veronii*	A600500	600500	Bangladesh	Case	3,177,970	479,873,470	106.9	44	209,513	4,488,340	58.79	1,731	SAMN14146682	SRR11247379	JAAKVD000000000
*Aeromonas veronii*	A600554	600554	Bangladesh	Case	3,709,180	560,086,180	124.7	36	237,609	4,490,252	58.84	1,751	SAMN14146686	SRR11247335	JAAKUZ000000000
*Aeromonas veronii*	A600709	600709	Bangladesh	Case	3,779,106	570,645,006	125.9	45	196,910	4,532,405	58.87	1,755	SAMN14146697	SRR11247280	JAAKUO000000000
*Aeromonas veronii*	A600715	600715	Bangladesh	Case	8,473,600	1,279,513,600	288.2	28	534,332	4,439,235	59.06	1,723	SAMN14146698	SRR11247279	JAAKUN000000000
*Aeromonas veronii*	A600717	600717	Bangladesh	Case	3,772,364	569,626,964	123.0	44	175,514	4,629,731	58.64	1,289	SAMN14147067	SRR11247300	JAAKWY000000000
*Aeromonas veronii*	A600782	600782	Bangladesh	Case	6,839,206	1,032,720,106	230.9	37	226,506	4,472,434	58.76	1,730	SAMN14146704	SRR11247273	JAAKUH000000000
*Aeromonas veronii*	A600987	600987	Bangladesh	Case	3,807,780	574,974,780	127.2	30	420,304	4,520,216	58.76	1,757	SAMN14146720	SRR11247255	JAAKTR000000000
*Aeromonas veronii*	A601031	601031	Bangladesh	Case	5,686,914	858,724,014	194.6	47	160,618	4,412,842	58.92	1,758	SAMN14146725	SRR11247510	JAAKTM000000000
*Aeromonas veronii*	A601046	601046	Bangladesh	Case	3,138,506	473,914,406	102.5	38	220,190	4,623,508	58.68	1,719	SAMN14146727	SRR11247507	JAAKTK000000000
*Aeromonas veronii*	A601074	601074	Bangladesh	Case	7,023,196	1,060,502,596	243.4	78	130,834	4,356,235	58.82	1,741	SAMN14146731	SRR11247503	JAAKTG000000000
*Aeromonas veronii*	A601116	601116	Bangladesh	Case	8,462,048	1,277,769,248	268.7	89	124,856	4,755,640	58.41	1,760	SAMN14146735	SRR11247499	JAAKTC000000000
*Aeromonas veronii*	A601117	601117	Bangladesh	Case	3,763,504	568,289,104	131.2	69	135,184	4,331,773	58.9	1,742	SAMN14146736	SRR11247498	JAAKTB000000000
*Aeromonas veronii*	A601125	601125	Bangladesh	Case	3,344,024	504,947,624	112.5	46	179,082	4,488,554	58.84	1,761	SAMN14146737	SRR11247496	JAAKTA000000000
*Aeromonas veronii*	A601158	601158	Bangladesh	Case	6,508,708	982,814,908	220.2	55	157,163	4,463,607	58.91	1,763	SAMN14146741	SRR11247492	JAAKSW000000000
*Aeromonas veronii*	A601264	601264	Bangladesh	Case	6,158,962	930,003,262	210.9	47	167,755	4,409,022	58.88	1,765	SAMN14146745	SRR11247488	JAAKSS000000000
*Aeromonas veronii*	A602296	602296	Bangladesh	Case	7,260,610	1,096,352,110	246.9	47	173,326	4,440,113	58.72	1,768	SAMN14146757	SRR11247472	JAAKSG000000000
*Aeromonas veronii*	A602535	602535	Bangladesh	Case	7,614,806	1,149,835,706	255.9	67	131,756	4,493,333	58.74	1,770	SAMN14146776	SRR11247454	JAAKRN000000000
*Aeromonas veronii*	A602602	602602	Bangladesh	Case	9,352,966	1,412,297,866	306.7	174	55,590	4,604,661	58.54	1,735	SAMN14146779	SRR11247450	JAAKRK000000000
*Aeromonas veronii*	A602650	602650	Bangladesh	Case	6,236,512	941,713,312	211.9	32	220,793	4,444,814	58.85	1,762	SAMN14146783	SRR11247446	JAAKRG000000000
*Aeromonas veronii*	A602909	602909	Bangladesh	Case	7,236,850	1,092,764,350	244.4	62	149,653	4,470,599	58.73	1,718	SAMN14146804	SRR11247423	JAAKQL000000000
*Aeromonas veronii*	A602927	602927	Bangladesh	Case	7,507,832	1,133,682,632	252.9	92	100,133	4,481,885	58.78	1,771	SAMN14146805	SRR11247250	JAAKQK000000000
*Aeromonas veronii*	A602992	602992	Bangladesh	Case	8,199,908	1,238,186,108	284.3	104	88,397	4,355,634	58.86	1,772	SAMN14146810	SRR11247244	JAAKQF000000000
*Aeromonas veronii*	A603020	603020	Bangladesh	Case	8,709,608	1,315,150,808	297.7	36	205,790	4,417,771	58.92	1,773	SAMN14146812	SRR11247242	JAAKQD000000000
*Aeromonas veronii*	A603323	603323	Bangladesh	Case	3,004,594	453,693,694	100.8	44	168,445	4,503,090	58.94	1,738	SAMN14147074	SRR11247293	JAAKWR000000000
*Aeromonas veronii*	A603369	603369	Bangladesh	Case	7,819,022	1,180,672,322	261.9	67	144,034	4,508,663	58.82	1,782	SAMN14146833	SRR11247219	JAAKPI000000000
*Aeromonas veronii*	A603378	603378	Bangladesh	Case	6,126,334	925,076,434	203.5	80	106,441	4,545,289	58.63	1,725	SAMN14146834	SRR11247218	JAAKPH000000000
*Aeromonas veronii*	A603596	603596	Bangladesh	Case	6,694,820	1,010,917,820	224.9	159	61,525	4,494,387	58.83	1,774	SAMN14146845	SRR11247206	JAAKOW000000000
*Aeromonas veronii*	A603609	603609	Bangladesh	Case	7,650,398	1,155,210,098	263.2	47	161,589	4,388,371	58.97	1,716	SAMN14146847	SRR11247202	JAAKOU000000000
*Aeromonas veronii*	A603640	603640	Bangladesh	Case	7,848,008	1,185,049,208	268.8	53	172,518	4,408,346	58.74	1,775	SAMN14146850	SRR11247199	JAAKOR000000000
*Aeromonas veronii*	A603651	603651	Bangladesh	Case	6,991,548	1,055,723,748	241.5	132	79,754	4,370,934	59.07	1,776	SAMN14146852	SRR11247197	JAAKOP000000000
*Aeromonas veronii*	A603672	603672	Bangladesh	Case	8,664,022	1,308,267,322	299.7	46	168,675	4,364,594	59.11	90	SAMN14146853	SRR11247196	JAAKOO000000000
*Aeromonas veronii*	A603760	603760	Bangladesh	Case	7,056,364	1,065,510,964	238.4	48	174,287	4,469,312	58.8	1,778	SAMN14146864	SRR11247184	JAAKOD000000000
*Aeromonas veronii*	A603946	603946	Bangladesh	Case	5,350,046	807,856,946	181.5	48	199,140	4,449,976	58.86	1,779	SAMN14146871	SRR11247176	JAAKNW000000000
*Aeromonas veronii*	A604208	604208	Bangladesh	Case	8,676,012	1,310,077,812	297.3	45	200,914	4,406,616	58.93	1,781	SAMN14146893	SRR11247150	JAAKNA000000000
*Aeromonas veronii*	A604277	604277	Bangladesh	Case	9,239,970	1,395,235,470	311.3	68	153,052	4,482,197	58.76	ND	SAMN14146896	SRR11247147	JAAKMX000000000
*Aeromonas veronii*	A604419	604419	Bangladesh	Case	7,651,586	1,155,389,486	255.5	42	161,758	4,521,853	58.69	1,724	SAMN14146909	SRR11247132	JAAKMK000000000
*Aeromonas veronii*	A604482	604482	Bangladesh	Case	7,445,982	1,124,343,282	241.7	49	168,419	4,651,075	58.52	1,721	SAMN14146915	SRR11247126	JAAKME000000000
*Aeromonas veronii*	A700007	700007	Pakistan	Case	5,796,612	875,288,412	191.3	37	188,116	4,574,526	58.33	1,784	SAMN14146926	SRR11247114	JAAKLT000000000
*Aeromonas veronii*	A700019	700019	Pakistan	Case	8,668,558	1,308,952,258	284.8	54	164,811	4,596,831	58.43	1,720	SAMN14146927	SRR11247112	JAAKLS000000000
*Aeromonas veronii*	A700201	700201	Pakistan	Case	7,235,132	1,092,504,932	242.1	74	143,793	4,511,790	58.73	443	SAMN14146936	SRR11247103	JAAKLJ000000000
*Aeromonas veronii*	A700590	700590	Pakistan	Case	7,309,058	1,103,667,758	241.9	93	117,629	4,563,013	58.6	1,787	SAMN14146955	SRR11247081	JAAKKQ000000000
*Aeromonas veronii*	A702133	702133	Pakistan	Case	4,490,350	678,042,850	150.2	42	220,804	4,515,330	58.8	1,788	SAMN14146971	SRR11247407	JAAKKA000000000
*Aeromonas veronii*	A702733	702733	Pakistan	Case	5,274,656	796,473,056	175.1	128	78,187	4,548,149	58.69	1,733	SAMN14146994	SRR11247382	JAAKJD000000000
*Aeromonas veronii*	A702865	702865	Pakistan	Case	7,049,856	1,064,528,256	238.3	67	142,211	4,466,383	58.82	1,789	SAMN14146997	SRR11247378	JAAKJA000000000
*Aeromonas veronii*	A703012	703012	Pakistan	Case	7,901,968	1,193,197,168	262.3	212	50,617	4,549,084	58.64	1,790	SAMN14147002	SRR11247373	JAAKIV000000000
*Aeromonas veronii*	A703332	703332	Pakistan	Case	7,400,130	1,117,419,630	252.7	54	165,022	4,421,511	58.84	1,791	SAMN14147021	SRR11247352	JAAKIC000000000
*Aeromonas veronii*	A703427	703427	Pakistan	Case	7,463,834	1,127,038,934	246.5	177	53,095	4,572,562	58.53	1,792	SAMN14147025	SRR11247348	JAAKHY000000000
*Aeromonas veronii*	A703499	703499	Pakistan	Case	7,355,114	1,110,622,214	251.3	62	146,268	4,420,253	58.81	1,742	SAMN14147028	SRR11247344	JAAKHV000000000
*Aeromonas veronii*	A703986	703986	Pakistan	Case	6,934,402	1,047,094,702	233.7	33	245,577	4,480,578	58.63	1,717	SAMN14147043	SRR11247328	JAAKHG000000000
*Aeromonas veronii*	A704022	704022	Pakistan	Case	8,629,200	1,303,009,200	294.2	58	156,403	4,428,995	58.88	1,749	SAMN14147046	SRR11247325	JAAKXT000000000
*Aeromonas veronii*	A704153	704153	Pakistan	Case	6,255,042	944,511,342	212.0	111	82,922	4,455,678	58.81	1,740	SAMN14147051	SRR11247318	JAAKXO000000000
*Aeromonas veronii*	A600282	600282	Bangladesh	Control	6,173,342	932,174,642	207.2	42	169,090	4,499,628	58.79	1,694	SAMN14146668	SRR11247192	JAAKVR000000000
*Aeromonas veronii*	A600527	600527	Bangladesh	Control	6,597,616	996,240,016	214.5	46	228,906	4,644,909	58.33	1,682	SAMN14146685	SRR11247346	JAAKVA000000000
*Aeromonas veronii*	A600584	600584	Bangladesh	Control	10,819,512	1,633,746,312	355.5	47	222,059	4,595,303	58.39	1,682	SAMN14146692	SRR11247286	JAAKUT000000000
*Aeromonas veronii*	A600886	600886	Bangladesh	Control	6,690,182	1,010,217,482	222.8	54	133,036	4,533,980	58.53	1,697	SAMN14146710	SRR11247266	JAAKUB000000000
*Aeromonas veronii*	A601019	601019	Bangladesh	Control	5,855,072	884,115,872	198.5	56	164,751	4,454,877	58.62	1,706	SAMN14146722	SRR11247253	JAAKTP000000000
*Aeromonas veronii*	A601126	601126	Bangladesh	Control	7,508,170	1,133,733,670	256.3	42	174,960	4,422,853	58.62	1,700	SAMN14146738	SRR11247495	JAAKSZ000000000
*Aeromonas veronii*	A602200	602200	Bangladesh	Control	6,511,792	983,280,592	220.4	34	211,483	4,461,723	58.78	1,683	SAMN14146752	SRR11247478	JAAKSL000000000
*Aeromonas veronii*	A602396	602396	Bangladesh	Control	7,823,504	1,181,349,104	261.1	34	225,489	4,523,979	58.72	1,681	SAMN14146760	SRR11247469	JAAKSD000000000
*Aeromonas veronii*	A600360	600360	Bangladesh	Control	6,323,132	954,792,932	213.4	52	168,488	4,474,007	58.84	1,747	SAMN14146675	SRR11247113	JAAKVK000000000
*Aeromonas veronii*	A600563	600563	Bangladesh	Control	7,922,504	1,196,298,104	279.7	76	123,221	4,276,388	58.85	1,752	SAMN14146688	SRR11247312	JAAKUX000000000
*Aeromonas veronii*	A600569	600569	Bangladesh	Control	7,232,104	1,092,047,704	242.5	40	231,986	4,503,215	58.77	1,753	SAMN14146689	SRR11247301	JAAKUW000000000
*Aeromonas veronii*	A600570	600570	Bangladesh	Control	6,264,068	945,874,268	214.7	45	167,625	4,404,737	58.92	1,754	SAMN14146690	SRR11247290	JAAKUV000000000
*Aeromonas veronii*	A600799	600799	Bangladesh	Control	7,561,986	1,141,859,886	252.8	33	294,451	4,516,701	58.77	1,756	SAMN14146705	SRR11247272	JAAKUG000000000
*Aeromonas veronii*	A600830	600830	Bangladesh	Control	7,258,760	1,096,072,760	252.9	82	108,940	4,334,285	58.8	1,734	SAMN14146707	SRR11247269	JAAKUE000000000
*Aeromonas veronii*	A601069	601069	Bangladesh	Control	5,738,164	866,462,764	184.2	37	206,081	4,704,462	58.67	1,759	SAMN14146730	SRR11247504	JAAKTH000000000
*Aeromonas veronii*	A601210	601210	Bangladesh	Control	6,415,266	968,705,166	209.8	40	202,016	4,617,546	58.49	1,764	SAMN14146743	SRR11247490	JAAKSU000000000
*Aeromonas veronii*	A602021	602021	Bangladesh	Control	6,382,136	963,702,536	219.0	26	442,720	4,400,024	59.07	1,766	SAMN14146750	SRR11247480	JAAKSN000000000
*Aeromonas veronii*	A602228	602228	Bangladesh	Control	5,929,196	895,308,596	200.2	71	140,811	4,472,981	58.71	1,767	SAMN14146754	SRR11247476	JAAKSJ000000000
*Aeromonas veronii*	A602240	602240	Bangladesh	Control	7,908,016	1,194,110,416	266.4	116	81,570	4,481,579	58.94	1,739	SAMN14146755	SRR11247475	JAAKSI000000000
*Aeromonas veronii*	A602456	602456	Bangladesh	Control	7,668,868	1,157,999,068	262.4	46	181,413	4,413,489	58.99	1,728	SAMN14146768	SRR11247460	JAAKRV000000000
*Aeromonas veronii*	A602459	602459	Bangladesh	Control	7,382,964	1,114,827,564	249.8	40	167,761	4,463,712	58.96	1,769	SAMN14146769	SRR11247459	JAAKRU000000000
*Aeromonas veronii*	A603395	603395	Bangladesh	Control	9,105,412	1,374,917,212	306.7	40	210,751	4,483,442	58.79	1,726	SAMN14146836	SRR11247216	JAAKPF000000000
*Aeromonas veronii*	A603551	603551	Bangladesh	Control	7,709,612	1,164,151,412	265.9	53	182,560	4,378,579	58.68	1,732	SAMN14146840	SRR11247211	JAAKPB000000000
*Aeromonas veronii*	A603571	603571	Bangladesh	Control	8,480,242	1,280,516,542	282.5	57	165,054	4,532,234	58.69	1,722	SAMN14146841	SRR11247210	JAAKPA000000000
*Aeromonas veronii*	A603737	603737	Bangladesh	Control	7,735,650	1,168,083,150	262.3	34	228,760	4,452,424	58.95	1,739	SAMN14146859	SRR11247189	JAAKOI000000000
*Aeromonas veronii*	A603748	603748	Bangladesh	Control	7,091,624	1,070,835,224	236.2	39	185,077	4,533,717	58.63	1,777	SAMN14146861	SRR11247187	JAAKOG000000000
*Aeromonas veronii*	A604273	604273	Bangladesh	Control	8,945,574	1,350,781,674	288.6	54	152,244	4,680,327	58.71	1,743	SAMN14146895	SRR11247148	JAAKMY000000000
*Aeromonas veronii*	A604418	604418	Bangladesh	Control	6,480,396	978,539,796	218.7	43	164,000	4,474,347	58.84	1,747	SAMN14146908	SRR11247133	JAAKML000000000
*Aeromonas veronii*	A604451	604451	Bangladesh	Control	6,633,746	1,001,695,646	226.1	55	159,425	4,429,888	58.84	1,783	SAMN14146913	SRR11247128	JAAKMG000000000
*Aeromonas veronii*	A604486	604486	Bangladesh	Control	8,837,048	1,334,394,248	302.8	43	216,943	4,406,760	59.04	1,729	SAMN14146916	SRR11247125	JAAKMD000000000
*Aeromonas veronii*	A700075	700075	Pakistan	Control	5,431,030	820,085,530	181.7	36	222,776	4,512,191	58.95	1,785	SAMN14146931	SRR11247108	JAAKLO000000000
*Aeromonas veronii*	A700550	700550	Pakistan	Control	6,283,994	948,883,094	216.3	88	108,897	4,385,888	58.99	1,786	SAMN14146953	SRR11247083	JAAKKS000000000
*Aeromonas veronii*	A703913	703913	Pakistan	Control	7,391,968	1,116,187,168	245.9	57	156,406	4,538,774	58.87	1,736	SAMN14147041	SRR11247330	JAAKHI000000000
*Aeromonas veronii*	A704038	704038	Pakistan	Control	6,914,628	1,044,108,828	235.8	54	164,645	4,428,809	58.94	1,793	SAMN14147047	SRR11247322	JAAKXS000000000

Given the paucity of sequenced human-derived *Aeromonas* spp. isolates, this collection of genomes will serve future studies as representative references.

## Data Availability

All data have been released and can be found in the associated SRA and genome assembly accession numbers listed in Table 1.
